# Comparative Reliability Studies and Analysis of Au, Pd-Coated Cu and Pd-Doped Cu Wire in Microelectronics Packaging

**DOI:** 10.1371/journal.pone.0078705

**Published:** 2013-11-07

**Authors:** Gan Chong Leong, Hashim Uda

**Affiliations:** Institute of Nano Electronic Engineering (INEE), Universiti Malaysia Perlis, Kangar, Perlis, Malaysia; RMIT University, Australia

## Abstract

This paper compares and discusses the wearout reliability and analysis of Gold (Au), Palladium (Pd) coated Cu and Pd-doped Cu wires used in fineline Ball Grid Array (BGA) package. Intermetallic compound (IMC) thickness measurement has been carried out to estimate the coefficient of diffusion (D_o_) under various aging conditions of different bonding wires. Wire pull and ball bond shear strengths have been analyzed and we found smaller variation in Pd-doped Cu wire compared to Au and Pd-doped Cu wire. Au bonds were identified to have faster IMC formation, compared to slower IMC growth of Cu. The obtained weibull slope, β of three bonding wires are greater than 1.0 and belong to wearout reliability data point. Pd-doped Cu wire exhibits larger time-to-failure and cycles-to-failure in both wearout reliability tests in Highly Accelerated Temperature and Humidity (HAST) and Temperature Cycling (TC) tests. This proves Pd-doped Cu wire has a greater potential and higher reliability margin compared to Au and Pd-coated Cu wires.

## Introduction

Recently, copper (Cu) wirebonding is widely deployed in microelectronics packaging. There are many copper metallurgies used which includes bare Cu wire, Pd-coated copper wire and Pd-doped Cu wire. The introduction of Pd element in bare Cu wire either by doping or coating is believed to increase the moisture reliability of Cu wirebonding in microelectronic packaging. Unlike conventional gold (Au) wirebonding, cu wirebonding faced bigger reliability and assembly challenges especially in extended reliability. Copper wirebonding appears to be the alternate materials and various engineering studies on copper wire development have been reported [1]–[4]. Zheng *et al.* reported the corrosion kinetics of various Al-metal IMCs on the basis of the cation transport model via the calculation of the chemical potentials of aluminum in IMCs. They established a guideline to enhance bonding corrosion resistance, improving the thermal stability of IMCs and suppressing the Al vacancy diffusion in Al_2_O_3_ layer by doping highly charged element(s) in Al pad [5]. Technical barriers and reliability challenges of Cu wirebonding in microelectronics packaging are well-identified [6]–[9]. Cu ball bond is more susceptible to moisture corrosion compared to gold ball bonds and undergo different corrosion mechanisms in microelectronic packaging [6], [10]. Gan *et al.* conducted studies on effects of bonding wires on unbiased HAST (UHAST) and TC reliability and found Cu with superior UHAST reliability compare to Au wire [10]–[12]. The interdiffusion between Au and Al across a thermally exposed Au-Al ball bond causes works done to compare the wearout reliability of various bonding wires in microelectronics packaging. McPherson has laid out the time-to-failure modeling in the reliability engineering works [13] which we applied in our reliability assessment of Au, Pd-coated Cu and Pd-doped Cu wires. Previous reliability characterizations have been conducted in comparing Au and Pd-coated Cu wires [14]–[17] but not on the newer Pd-doped Cu wire. Kouters *et al.* discovered the Cu-rich intermetallics Cu_9_Al_4_ and Cu_3_Al_2_ are less sensitive to fracture than the Al-rich intermetallics. However, these Cu-rich intermetallics have a smaller atomic volume and will show a volumetric shrinkage during formation. This is expected to cause a large internal stress during thermal aging [18]. Lassnig *et al.* also developed a fast mechanical shear fatigue test technique for the quality assessment of thermosonic ball bonded interconnects was developed to estimate their lifetime behavior [19]. Hence, extended reliability is crucial to determine the lifetime of gold and copper ball bonds (Pd-coated or Pd-doped) in microelectronics packaging. The motivation of this study is to apply wearout lifetime study on newer Pd-doped Cu wire and compare the post stresses IMC thicknesses, wire pull strength and ball bond shear strength to conventional Au and Pd-coated Cu wires.

## Wire Construction Analysis and Experimental Matrix

### Materials and Preparation

The key materials used include 0.8 mil Pd-coated Cu wire and 4N (99.99% purity) Au wire, fine pitch BGA package, 110 nm device which to be packed in fortified Fineline BGA package, green (<20 ppm Chloride in content) in molding compound and substrate. All direct material used in this evaluation study for the 110 nm, low-k device (with top Al metallization bondpad) for packaging purpose. For the Intermetallic Compound (IMC) measurements, 45 units of Au, Pd-coated Cu and Pd-doped Cu wires bonded on Fine pitch 64-ball BGA packages are subjected for High Temperature Storage [20] at 150°C, 175°C and 200°C aging temperatures. IMC thicknesses were measured by using Secondary electron microscope to calculate the coefficient of diffusion, D_o_ and activation energies of FBGA64 package with different sets of packaging materials. Wire pull and ball bond shear tests were carried out on post HAST, TC samples at extended readouts for first ball bond integrity check. In this study, there are total 9 legs comprising of Pd-coated Cu wire and 4N Au wire bonded on Fine pitch 64-ball BGA packages on a 2L substrate. Sample size used is 80 units for each stresses. The corresponding stress tests and its conditions are tabulated in Table 1. After electrical test, good samples were then subjected for Preconditioning and 3 times reflow at 260°C as described in JEDEC IPC-STD 020 standard, followed by unbiased HAST stress testing per JESD22A-110D at 110°C/85%RH, 3.6V [21], biased HAST stress and temperature cycling tests per JESD22A-104D at −40°C to 150°C [22]. Electrical testing was conducted after each hours and cycles of stress to check Au and PdCu ball bond integrity in terms of its moisture and thermo-mechanical reliability with various conditions. The units are stressed until wearout failures and reliability plots are analyzed.

**Table 1:** Summary of experimental matrix.

Wire Type	Test Type	Test Conditions	SampleSize (units)
Au	TC	−40°C to 150°C	80
Au	Biased HAST	85%RH, 110°C	80
Au	HTSL	150°C, 175°C, 200°C	45
Pd-coated Cu	TC	−40°C to 150°C	80
Pd-coated Cu	Biased HAST	85%RH, 110°C	80
Pd-coated Cu	HTSL	150°C, 175°C, 200°C	45
Pd-doped Cu	TC	−40°C to 150°C	80
Pd-doped Cu	Biased HAST	85%RH, 110°C	80
Pd-doped Cu	HTSL	150°C, 175°C, 200°C	45

## Results

### IMC Growth Kinetics of Au, Pd-Coated Cu and Pd-Doped Cu Ball Bonds

IMC diffusion coefficients, *D* of Au, Pd-coated Cu and Pd-doped Cu ball bond were determined from the IMC thicknesses over aging time at different elevated temperatures as shown in Table 2. The average IMC thicknesses are tabulated in Table 2 for different types of ball bonds. It clearly indicates the Au ball bond exhibits higher IMC growth rate compared to Pd-coated and Pd-coped Cu ball bonds. This is notable as Au ball bonds are always found with higher Au atom diffusion into Al bondpad after long duration of aging tests [1], [10], [11]. The coefficient of IMC diffusion (in unit m^2^s^−1^) is calculated by using Eq.1.
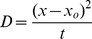
(1)Where *D* = coefficient of IMC diffusion (in unit m^2^s^−1^). = growth of IMC thicknesses over time (in unit m).*x_o_* = initial IMC thickness at time zero.*x* = IMC thickness at aging time, t. = aging time (in unit seconds).

**Table 2:** IMC Diffusion Kinetics of Au and Cu wires used in 110 nm device.

Wire Type	Test Type	Test Conditions	Average IMC thicknesses (um)	*D* (Average) m^2^s^−1^
Au	HTSL	150°C	2.91	8.08E-20
Au	HTSL	175°C	3.10	9.33E-19
Au	HTSL	200°C	3.61	1.11E-18
Pd-coated Cu	HTSL	150°C	0.46	9.63 E-22
Pd-coated Cu	HTSL	175°C	0.48	2.08E-21
Pd-coated Cu	HTSL	200°C	0.49	2.02E-20
Pd-doped Cu	HTSL	150°C	0.52	6.78E-21
Pd-doped Cu	HTSL	175°C	0.56	5.86E-20
Pd-doped Cu	HTSL	200°C	0.58	2.18E-19

### IMC Growth Analysis and Plotting

**Figure 1:**
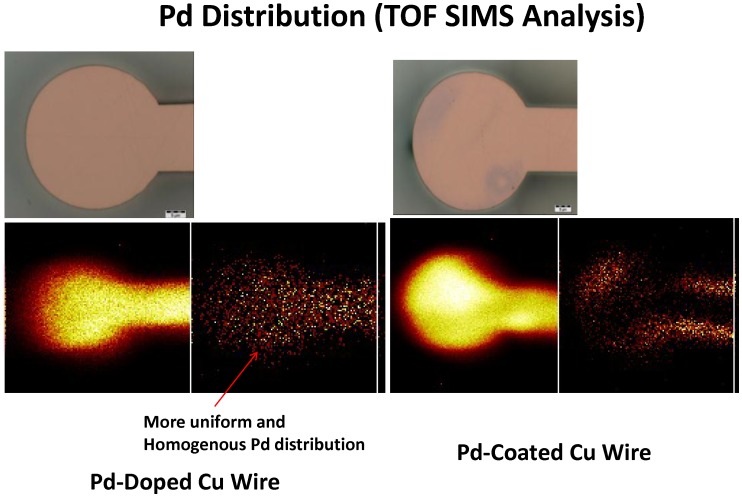
Wire constructions of Pd-coated Cu and Pd-doped Cu wires used in 110 nm device. TOF-SIM results show more homogenous Palladium distribution in Pd-doped Cu wire compared to Pd-coated Cu wire in Free Air Ball Formation (FAB).

**Figure 2:**
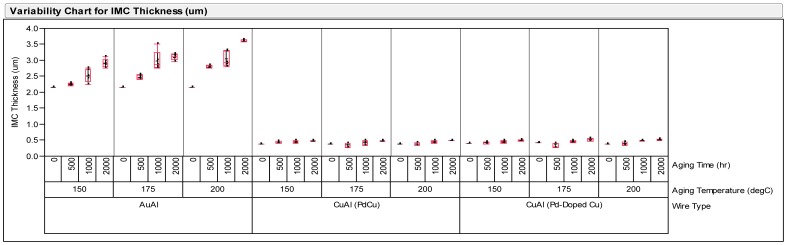
IMC thickness against aging time plotting of three wire types used in 110 nm device FBGA 64 package.

Figure 1 shows the TOF-SIMS results of Pd-coated Cu ball bond and Pd-doped Cu ball bonds. More homogenous Palladium distribution in Pd-doped Cu wire compared to Pd-coated Cu wire in Free Air Ball Formation (FAB). IMC growth analysis is carried out by plotting the Pd-coated and Pd-doped CuAl and AuAl IMC thicknesses (in mm or µm) against time. Cu wire is well-known to be less IMC growth rate if compared to Au ball bond onto Al metallization [1], [10], [11]. Au ball bond interfacial fracture is one of the bonding failures attributed to interface AuAl IMC oxidation and Kirkendall micro-voiding [1], [11]. Figure 2 indicates the IMC thickness against high temperature aging time after 150°C, 175°C and 200°C aging. In our evaluation, we observed thinner CuAl IMC layer formation of Pd-coated Cu and Pd-doped Cu ball bonds than in Au ball bonds.

### Wire Pull and Ball Bond Shear Strength Post Reliability Stresses

**Figure 3:**
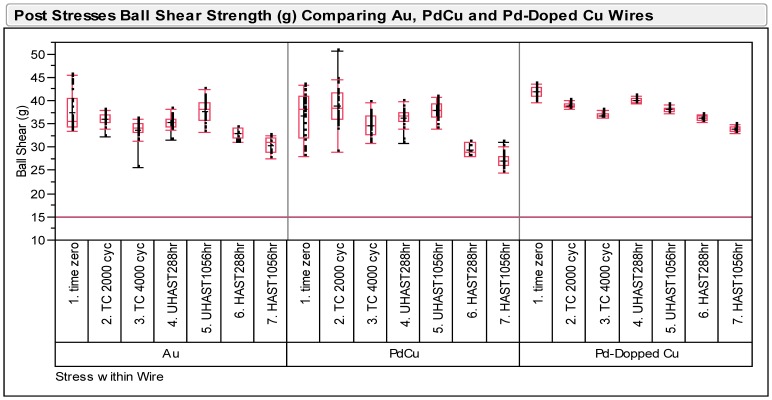
Post stresses ball shear strengths (g) of Au, Pd-coated Cu and Pd-doped Cu wires.

**Figure 4:**
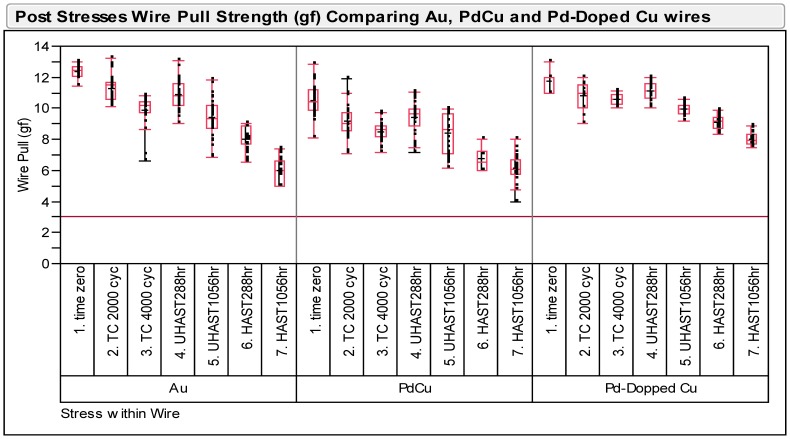
Post stresses wire pull strengths (g) of Au, Pd-coated Cu and Pd-doped Cu wires.

**Figure 5:**
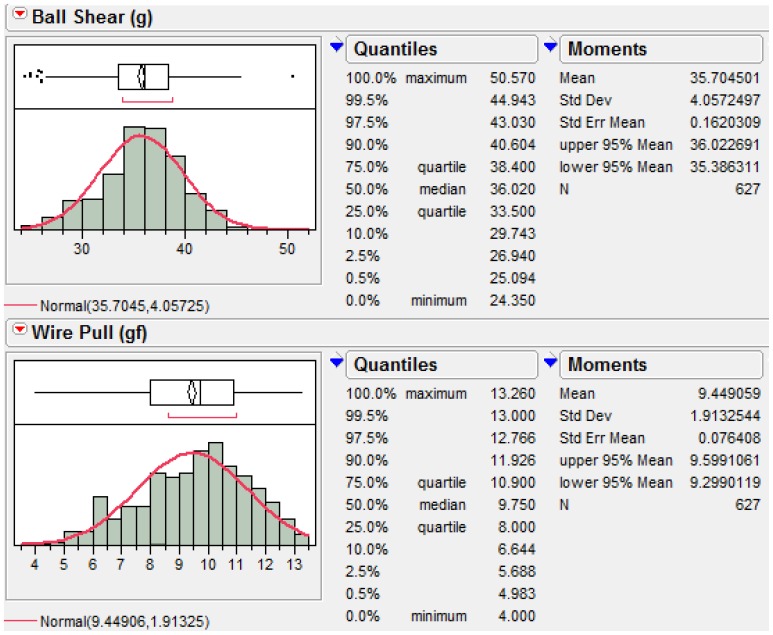
Distribution analysis of ball shear and wire pull data.

Wire pull and ball shear tests have been conducted on three types of ball bonds after long term reliability stresses of biased HAST, unbiased HAST and TC tests. The minimum passing criteria of wire pull and ball shear strengths are at 3 gf and 15 g respectively. Figure 3 shows variability plot compares ball shear values after various reliability stresses and Pd-doped Cu ball bonds show less variation in its ball shear values compared to Au and Pd-coated Cu wire. The degree of degradation of Pd-doped Cu ball bonds are lesser compared to Au and Pd-coated Cu ball bonds. Similarly, we observed Pd-doped Cu ball bonds exhibits lesser degradation in its wire pull strength in variability plot (as shown in Figure 4). Three ball bonds types are far exceeded the minimum required wire pull strength value of 3 gf and ball shear value of 15 g respectively. Statistical normality test has been carried out to examine the wire pull and ball shear strength values and both distributions are well-fitted to Normal distribution (as indicated in Figure 5). Table 3 tabulates the post reliability stresses ball shear summary and Table 4 tabulates the post reliability stresses wire pull strength of three ball bond types.

**Table 3:** Ball Shear Strength Summary.

Wire Type	Test Type	Leg	Mean ball shear strength (g)	Standard deviation
Au	Fresh units	Time zero	37.34	3.93
Au	TC	2000 cycles	35.92	1.22
Au	TC	4000 cycles	33.68	1.93
Au	UHAST	288 hour	35.17	1.22
Au	UHAST	1056 hour	37.64	2.45
Au	HAST	288 hour	32.80	1.13
Au	HAST	1056 hour	30.42	1.36
Pd-coated Cu	Fresh units	Time zero	36.78	4.96
Pd-coated Cu	TC	2000 cycles	37.87	4.26
Pd-coated Cu	TC	4000 cycles	34.67	2.32
Pd-coated Cu	UHAST	288 hour	36.30	1.84
Pd-coated Cu	UHAST	1056 hour	36.83	1.77
Pd-coated Cu	HAST	288 hour	29.26	1.23
Pd-coated Cu	HAST	1056 hour	27.06	1.54
Pd-doped Cu	Fresh units	Time zero	41.84	1.05
Pd-doped Cu	TC	2000 cycles	38.92	0.44
Pd-doped Cu	TC	4000 cycles	36.73	0.50
Pd-doped Cu	UHAST	288 hour	40.08	0.59
Pd-doped Cu	UHAST	1056 hour	38.02	0.51
Pd-doped Cu	HAST	288 hour	36.09	0.59
Pd-doped Cu	HAST	1056 hour	33.86	0.60

**Table 4:** Wire pull strength summary.

Wire Type	Test Type	Leg	Mean Wire pull strength (gf)	Standard deviation
Au	Fresh units	Time zero	12.38	0.39
Au	TC	2000 cycles	11.31	0.84
Au	TC	4000 cycles	9.88	0.96
Au	UHAST	288 hour	10.89	0.97
Au	UHAST	1056 hour	9.38	1.25
Au	HAST	288 hour	8.04	0.72
Au	HAST	1056 hour	9.38	1.25
Pd-coated Cu	Fresh units	Time zero	10.52	0.99
Pd-coated Cu	TC	2000 cycles	9.18	1.06
Pd-coated Cu	TC	4000 cycles	8.48	0.64
Pd-coated Cu	UHAST	288 hour	9.43	1.04
Pd-coated Cu	UHAST	1056 hour	8.37	1.27
Pd-coated Cu	HAST	288 hour	6.73	0.83
Pd-coated Cu	HAST	1056 hour	6.16	0.90
Pd-doped Cu	Fresh units	Time zero	11.77	0.73
Pd-doped Cu	TC	2000 cycles	10.85	0.77
Pd-doped Cu	TC	4000 cycles	10.60	0.34
Pd-doped Cu	UHAST	288 hour	11.12	0.59
Pd-doped Cu	UHAST	1056 hour	9.94	0.35
Pd-doped Cu	HAST	288 hour	9.09	0.45
Pd-doped Cu	HAST	1056 hour	8.01	0.37

### Biased HAST and TC Wearout Reliability Analysis

The Weibull distribution is a weakest-link type distribution. By using the term weakest link, one means that the failure of the whole (for example a chain) is dominated by the degradation rate for the weakest element (one of the links). The Weibull distribution is also very useful for system reliability where the entire system fails when one of the constituent components fails. The Weibull probability density function is defined by Eq. 2 [13].



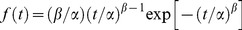
(2)


Unlike the lognormal distribution (where the cumulative failure probability F(t) must be obtained by numerical methods represented by the error function), an analytical expression can be found for the cumulative Weibull failure probability function, as in Eq. 3



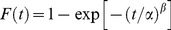
(3)


Rearranging Eq. (3) and taking the appropriate logarithms, one obtains Eq. 4.



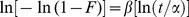
(4)where Weibull characteristic time as: *α* refers to t_63.2_ and β refers to the weibull slope and F is the failure rate. An alternative method for performing the Weibull plotting is through the use of Weibit. The conversion of cumulative fraction failed *F* into Weibit is given in Eq. 5:



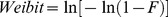
(5)


**Figure 6:**
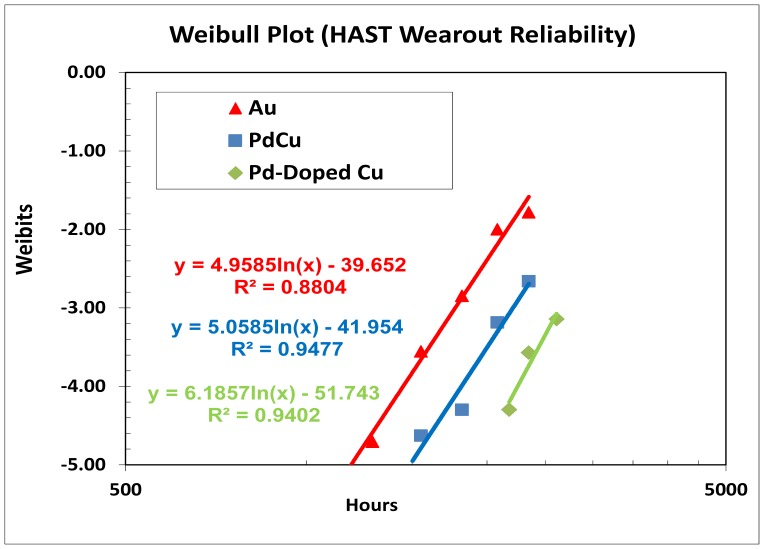
Biased HAST weibull-fitted wearout reliability plot.

**Figure 7:**
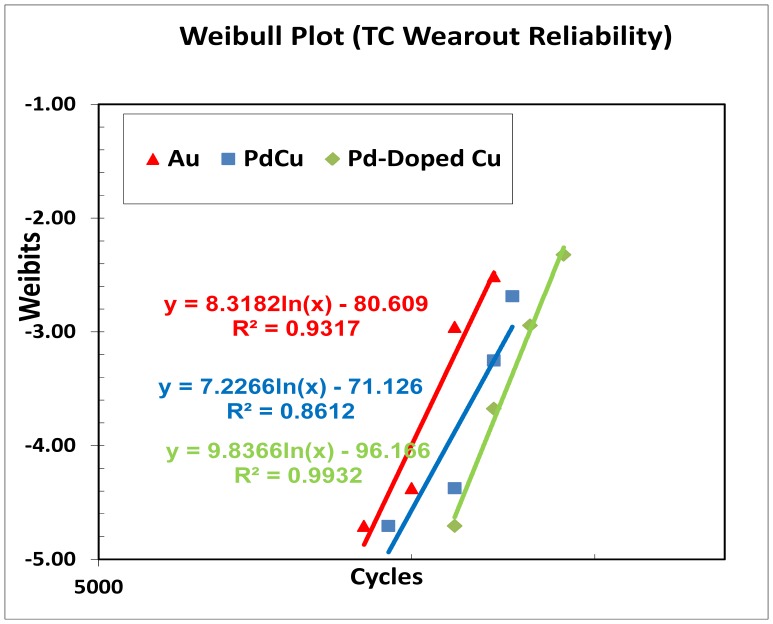
TC weibull-fitted wearout reliability plot.

The three samples assembled with Au, Pd-coated Cu and Pd-doped Cu wires were subjected to wearout reliability stresses such as biased HAST and TC tests. Parts are stressed until the cumulative 50% of the total sample reached failures. Table 5 tabulates the key biased HAST and TC wearout reliability parameters included first failure (t_first_), mean-time-to failure (t_50_), and characteristic life (t_63.2_). Figures 6 and 7 show biased HAST and TC weibull reliability plots of three types ball bonds respectively and the key weibull parameters of three wire types of biased HAST and TC stresses are tabulated in Table 5.

**Table 5:** Wearout Reliability of biased HAST and TC for Three Ball Bonds.

Wire Type	Test Type	Test Conditions	t_first_(hr/cyc)	t_50_	t_63.2_ (η)	ß
Au	TC	−40°C to150°C	9000	15060	15691	8.32
Au	HAST	85%RH,110°C	1289	2967	3189	4.96
Pd-coated Cu	TC	−40°C to150°C	9500	16756	17504	7.23
Pd-coated Cu	HAST	85%RH,110°C	1553	3593	3848	5.06
Pd-doped Cu	TC	−40°C to150°C	11000	16932	17570	9.84
Pd-doped Cu	HAST	85%RH,110°C	2177	4489	4774	6.19

## Discussion

We found Pd-doped Cu ball bonds exhibits similar CuAl IMC thickness growth rate with respect to Pd-coated Cu ball bonds. For Pd-doped CuAl IMC, it is pretty similar but thicker IMC grown compared to Pd-coated CuAl IMC. This is mainly attributed to the homogenous Pd element doped into Cu free air ball prior to the thermosonic wirebonding. Heraues wire vendor compares Pd distribution in both Pd-doped and Pd-coated Cu wires and found uniform Pd distribution is observed within RelCu Free Air Ball (FAB) on melting the wire [23]. Hui X *et al.* [24], [25] reported when a Pd coated copper wire is being heated and melted to form a FAB, the Pd layer does not completely dissolve into copper ball to form a total solid solution according to solid solubility of the two metals. This is because in actual melting process of the wire, the duration of heating is too short to allow complete dissolution of Pd into copper core material before cooling and the solidification rate is too fast in the FAB formation process due to the short electric flame off (EFO) firing time to melt the tip of wire and the use of process gas such as nitrogen/hydrogen (95%/5%) mixture or pure nitrogen to protect the ball from oxidation before bonding onto the IC pad. Coefficient of diffusion, *D* can be determined from Eq. 1 from the AuAl and CuAl IMC thicknesses growth over aging time under various aging temperatures. This could be due to the slower CuAl IMC diffusion rate compared to AuAl which is usually with at least 5x faster AuAl IMC growth rate and found Kirkendall micro voiding [1], [11]. Our previous works reveal Pd-coated Cu wire exhibits higher hours and cycles-to-failure than Au wire in biased HAST and TC wearout reliability studies [12], [14]–[15]. In this extended wearout reliability stress, Pd-doped Cu wire is found with higher hours-to-failure in biased HAST wearout reliability test which is fitted to Weibull distribution. It appears to be that Pd-doped Cu wire is the best leg in biased HAST and TC wearout reliability margin compared to conventional Au and Pd-coated Cu wire legs. This could be attributed to the homogenous Pd distribution in free air ball of Pd-doped Cu ball bond which inhibit CuAl IMC interfacial corrosion as reported by various researchers in bare Cu and Pd-coated Cu ball bonds [11]–[12].

## Conclusion

In this research, we analyzed the effects of wire alloy on CuAl and AuAl IMC diffusion kinetics, post stresses wire pull and ball bond shear degradation trends and HAST and TC wearout reliability results. Pd-doped Cu wire is observed with faster IMC growth compared to Pd-coated Cu wire but much slower than conventional Au wire. In our study, higher diffusion coefficient, *D* is found on Pd-doped Cu wire compared to Pd-coated Cu wire. Au wire is with the highest *D_._* This is notable as Cu slower CuAl IMC diffusion rate compared to AuAl which is usually with at least 5x faster AuAl IMC growth rate and found Kirkendall microvoiding [1], [11]. Pd-doped Cu wire shows lesser variation and more stable degradation trend in post stresses wire pull and ball bond shear strengths. Among the three bonding wires, Pd-doped Cu wire also demonstrates highest HAST and TC wearout reliability margins. Hence Pd-doped Cu wire will become one of the promising wire alloys in microelectronic packaging in the near future.
