# Correction: Comparative Reliability Studies and Analysis of Au, Pd-Coated Cu and Pd-Doped Cu Wire in Microelectronics Packaging

**DOI:** 10.1371/annotation/21a4e441-0c3f-4319-9833-91d316b99d43

**Published:** 2014-01-02

**Authors:** Gan Chong Leong, Hashim Uda

The name of the first author of the article is incorrect. The first author's correct name is: Chong Leong Gan. The correct Citation is: 

Chong Leong G, Uda H (2013) Comparative Reliability Studies and Analysis of Au, Pd-Coated Cu and Pd-Doped Cu Wire in Microelectronics Packaging. PLoS ONE 8(11): e78705. doi:10.1371/journal.pone.0078705. 

The correct abbreviation of the first author's name in the Author Contributions statement is: CLG. 

